# Impact of Different π-Bridges on the Photovoltaic Performance of A-D-D′-D-A Small Molecule-Based Donors

**DOI:** 10.3390/molecules29174231

**Published:** 2024-09-06

**Authors:** Lingjun Yang, Yu Wu, Pachaiyappan Murugan, Peng Liu, Yulong Peng, Zhiyong Qiu, Zaifang Li, Changlin Yu, Shiyong Liu

**Affiliations:** 1 Jiangxi Provincial Key Laboratory of Functional Molecular Materials Chemistry, Department of Chemistry and Chemical Engineering, Jiangxi University of Science and Technology, Ganzhou 341000, China; 17779114525@163.com (L.Y.); 18011263004@163.com (Y.W.); p.kumu29@gmail.com (P.M.); 6720221036@mail.jxust.edu.cn (P.L.); 6720230419@mail.jxust.edu.cn (Y.P.); 6720230416@mail.jxust.edu.cn (Z.Q.); 2 China-Australia Institute for Advanced Materials and Manufacturing (IAMM), Jiaxing University, Jiaxing 314001, China; 3 Guangdong Provincial Key Laboratory of Advanced Green Lubricating Materials, Maoming 525000, China; yuchanglinjx@163.com

**Keywords:** organic solar cells, thiophene π-bridges, DTBDT, A-D-D′-D-A type, Y6

## Abstract

Three small donor molecule materials (**S1**, **S2**, **S3**) based on dithiophene [2,3-d:2′,3′-d′]dithiophene [1,2-b:4,5-b′]dithiophene (DTBDT) utilized in this study were synthesized using the Vilsmeier–Haack reaction, traditional Stille coupling, and Knoevenagel condensation. Then, a variety of characterization methods were applied to study the differences in optical properties and photovoltaic devices among the three. By synthesizing **S2** using a thiophene π-bridge based on **S1**, the blue shift in ultraviolet absorption can be enhanced, the band gap and energy level can be reduced, the open circuit voltage (*V*_OC_) can be increased to 0.75 V using the **S2**:**Y6** device, and a power conversion efficiency (PCE) of 3% can be achieved. Also, after developing the device using **Y6**, **S3** introduced the alkyl chain of thiophene π-bridge to **S2**, which improved the solubility of tiny donor molecules, achieved the maximum short-circuit current (*J*_SC_ = 10.59 mA/cm^2^), filling factor (FF = 49.72%), and PCE (4.25%). Thus, a viable option for future design and synthesis of small donor molecule materials is to incorporate thiophene π-bridges into these materials, along with alkyl chains, in order to enhance the device’s morphology and charge transfer behavior.

## 1. Introduction

The power conversion efficiency (PCE) of single-junction polymer organic solar cells (PSCs) has been substantially enhanced in recent years due to the introduction of novel photovoltaic organic materials, particularly Y-series non-fullerene small molecule materials [1,2,3,4,5,6,7,8,9,10,11,12,13,14,15,16,17]. The industrialization of mass production has brought about a new challenge: even though polymer materials have an excellent process and high efficiency when it comes to creating membranes [18,19,20,21,22], because their chemical structure is not very apparent and their repeatability is poor, these problems in the manufacturing process can be better addressed by small molecules [23,24,25,26]. There has been significant advancement in the PCE of all small molecule organic solar cells (ASM OSCs) recently [27,28,29,30,31,32,33,34], but the performance of these cells is still inferior to that of PSCs. This is primarily because the donor and acceptor materials have a short and similar acceptor–donor–acceptor (A-D-A)-type molecular structure, which results in similar physical and chemical properties and makes it difficult to control the crystallinity of these materials. Because it is difficult for the blending membrane to create a nano-scale interpenetrating network structure, both short-circuit current (*J*_SC_) and filling factor (FF) are low.

An important component of the donor (D′) in the DTBDT unit—which stands for dithiophene [2,3-d:2′,3′-d′]dithiophene [1,2-b:4,5-b′]dithiophene—is capable of donating electrons and transporting carriers with remarkable efficiency [35,36,37,38,39,40,41,42,43,44]. By incorporating the 4-(2-ethylhexyl)-4H-dithieno [3,2-b:2′,3′-d]pyrrole (DTP46) into the molecular framework, the transport efficiency of carriers can be enhanced. It is well known that adding π-bridges to materials with tiny donor molecules changes their absorbance and crystallinity, and this is because DTP46’s stiff planar-conjugated structure promotes the coplanarity of molecules [45,46,47,48,49,50,51]. In its interaction with the central conjugated unit, 3-butyl-2-thioxothiazolidin-4-one (RDN-34) can form an A-D-A type structure that, thanks to its electron absorption capacity (A), has a superior intramolecular charge transfer effect (ICT) effect; this process can also prolong electron cloud delocalization [52,53,54,55,56,57,58,59,60,61,62]. By incorporating thiophene as a π-bridge into the small donor material, the *J*_SC_ and FF of organic solar cells (OSCs) can be enhanced, resulting in a larger PCE. Furthermore, via the addition of alkyl chains to the thiophene π-bridge, the π-π packing of the molecules can be enhanced, resulting in more compact layer packing and enhanced crystallization and aggregation of the material [63,64,65,66,67].

**Scheme 1:**
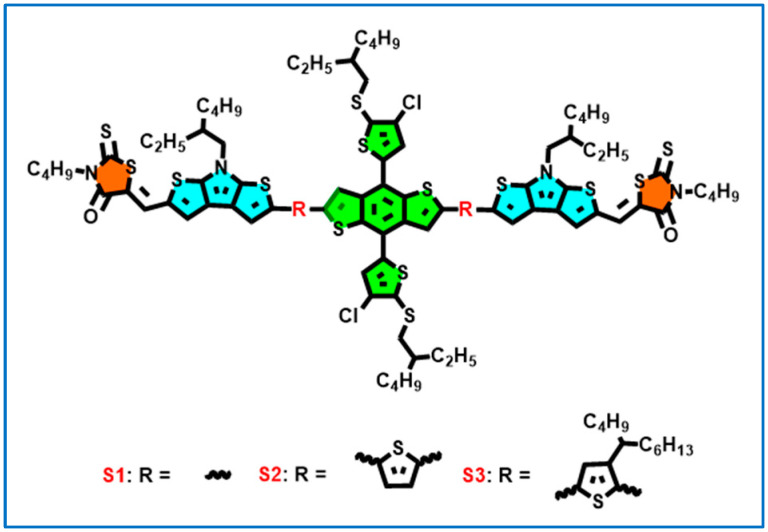
Structure of **S1**, **S2**, and **S3**.

This study details the successful creation of three tiny donor molecular materials based on DTBDT, designated as **S1**, **S2**, and **S3** (Scheme 1). **S2** is an example of this; it expands the initial conjugated plane by adding a thiophene π-bridge to **S1**, which is based on **S1**. In addition to improving charge transport and exciton ionization, this also leads to an improvement in the morphology of the composite film, which results in an increase in *J*_SC_ (8.08 mA/cm^2^ to 9.23 mA/cm^2^), FF (42.16% to 42.93%), and PCE (2.42% to 3%). A combination of an alkyl chain and the thiophene π-bridge allows for the production of **S3**, as **S1** and **S2** are poorly soluble in common organic solvents such as dichloromethane, trichloromethane, and chlorobenzene. When **S3** and acceptor molecule **Y6** are mixed, the film becomes free of insoluble particles. The device has several advantages, such as better carrier mobility, the ability to suppress bimolecular recombination, improved exciton dissociation and charge collection, and more balanced mobility. As a consequence of this, the **S3**:**Y6** devices exhibit an improved open circuit voltage (*V*_OC_ = 0.80 V), *J*_SC_ (10.64 mA/cm^2^), and FF (49.72%), while simultaneously succeeding in achieving an ideal PCE (4.25%).

## 2. Results and Discussion

### 2.1. Synthesis and Characterization

**Scheme 2:**
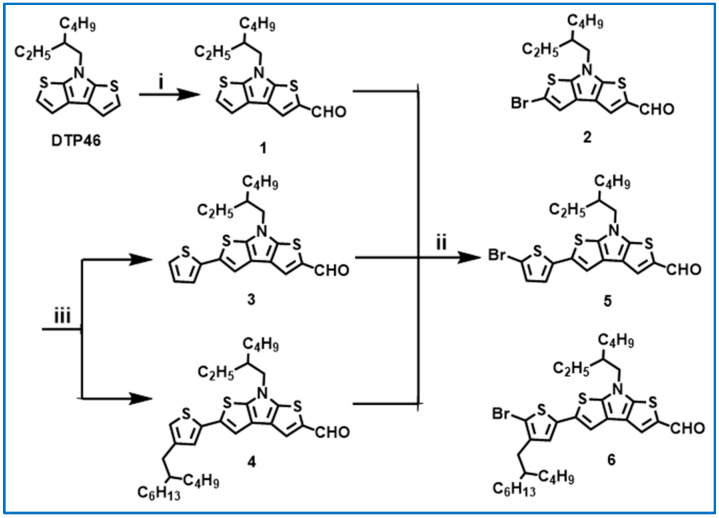
Synthesis of DTP46 precursor. (i) 1.2 eq DMF, 1.2 eq POCl_3_, 25.9 eq anhydrous DCE, 0 °C, 4 h; (ii) 1 eq NBS, anhydrous CHCl_3_, r.t.; (iii) 5% Pd(PPh_3_)_4_, anhydrous toluene, 110 °C, 48 h.

**Scheme 3:**
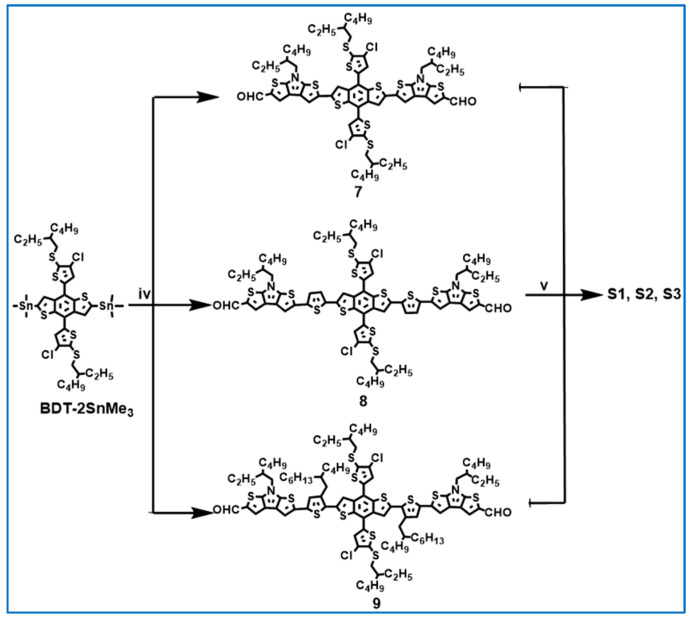
Synthesis of **S1**, **S2**, and **S3**. (iv) 5% Pd(PPh_3_)_4_, anhydrous toluene, 100 °C, 48 h; (v) anhydrous toluene, acetic anhydride, BF_3_-OEt_2_, r.t.

The three-part synthesis method of **S1**, **S2**, and **S3** is depicted in Scheme 2 and Scheme 3. Following the pre-synthesis of the DTP46 precursor (Scheme 2), the initial step was to produce an aldehyde intermediate 1 (DTP46-CHO) using the Vilsmeier–Haack reaction of DTP46. Subsequently, the DTP46-CHO was brominated with the N-bromosuccinimide (NBS) reagent, which resulted in the formation of intermediate 2 (Br-DTP46-CHO). Traditional Stille coupling is employed to synthesize aldehyde intermediates 3 and 4 with thiophene π-bridges in order to investigate the impact of thiophene π-bridges on small donor molecule materials. Derivatives 5 and 6 are produced by brominating the 3 or 4 using NBS reagent. Then, 7, 8, and 9 are formed by combining 2, 5, or 6 with BDT-2SnMe_3_, which is 4,8-(5-(2-(thio-2-ethylhexyl))4-chlor-othiophene-2-yl)-[1,2-b:4,5-b′]thiophene-[3,4-c]dithiobenzene, using the classic Stille coupling method followed by the target products **S1**–**S3** were synthesized by Knoevenagel condensation. **S1** and **S2** do not dissolve well in common solvents like dichloromethane, trichloromethane, chlorobenzene, and others because they have fewer alkyl chains. When mixed with acceptor **Y6**, the resulting film has small particles that cannot dissolve. **S3** was synthesized by replacing the original thiophene π-bridge with thiophene with an alkyl chain based on **S2**. This resulted in **S3** having good solubility in common solvents. The great purity of all these tiny donor molecule materials is confirmed by ^1^H NMR (Supplementary Information, Figures S1–S12).

### 2.2. Opto-Electronic Properties

**Figure 1:**
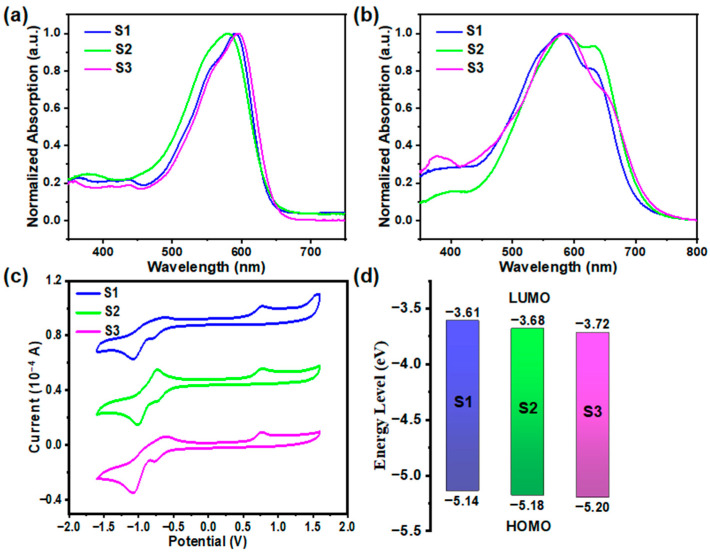
UV-*vis* absorption spectra of **S1**, **S2**, and **S3** (**a**) in CHCl_3_ solution and (**b**) in thin films; (**c**) CV curves; (**d**) FMO alignments.

Table 1 displays the detailed data, and Figure 1a,b displays the results of the UV-*vis* absorption spectra testing of three small donor molecules (**S1**, **S2**, and **S3**) in a CHCl_3_ solution and thin film. **S1**, **S2**, and **S3** display a broad and extensive absorption spectrum between 450 nm and 650 nm in a CHCl_3_ solution. The blue shift in **S2**’s ultraviolet absorption curves can be explained by the fact that the electron-donor central unit BDT-2SnMe_3_ and the peripheral weakly electron-absorbing RDN-34 terminal group have a weaker D-A interaction, as revealed by comparing the UV absorption curves of **S1** and **S2**. The addition of alkyl chains to the thiophene π-bridge enhances the D-A effect between groups and the degree of molecular chain order. As a result, **S3** exhibits a slight redshift phenomenon and a similar absorption trend to **S1** [68]. This phenomenon is further supported by the A_0-0_/A_0-1_ ratio, which measures the relative sizes of the right and left acromion, and the A_0-0_/A_0-1_ ratio of **S2** is smaller compared to other small donor molecules. The addition of the thiophene π-bridge reduces the strength of the D-A effect between the groups, as indicated by the weak π-π interaction between the chains. With the introduction of thiophene π-bridge, the optical bandgaps of **S1**, **S2**, and **S3** gradually narrow (1.75 to 1.69 eV) in Table 1. In solid films, **S1**, **S2**, and **S3** exhibit an absorption range that is wider and wider than in solution (430–720 nm). Furthermore, the maximum absorption is red-shifted by more than 90 nm in comparison to the solution. This is typically the result of enhanced main chain intermolecular interactions of small donor molecules and stronger aggregation effects in condensed matter.

The cyclic voltammetry (CV) technique was utilized in order to determine the frontier molecular orbital (FMO) levels of **S1**, **S2**, and **S3**, as can be seen in Figure 1c. In Figure 1d, it is demonstrated that the addition of a thiophene π-bridge lowers the highest occupied molecular orbital (HOMO) level of small donor molecule materials (−5.14 to −5.18 eV). The addition of alkyl chains to the thiophene π-bridge further lowers the HOMO level (−5.18 to −5.20 eV). As a result of this gradual decrease in HOMO level, the *V*_OC_ of **S1**, **S2**, and **S3** gradually increases (0.71–0.80 V). There is not a significant difference between the HOMO energy levels of **S1**, **S2**, and **S3** due to the fact that all three of these atoms share the same weak electron-absorbing end group, which is denoted by RDN-34.

### 2.3. Photovoltaic Performances

**Figure 2:**
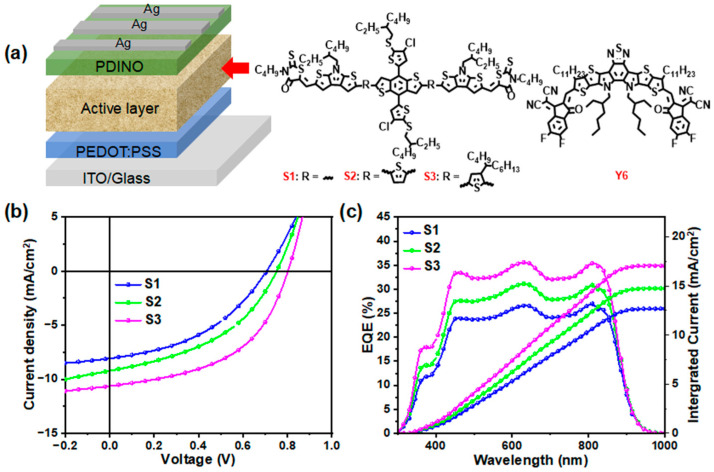
(**a**) Schematic device structure and chemical structures of **S1**, **S2**, **S3**, and **Y6**; (**b**) *J-V* curves; (**c**) corresponding EQE spectra of the OPVs.

During the process of evaluating the photovoltaic performance, the OSCs device was built with a structure consisting of ITO/PEDOT:PSS/Donor:Y6/PDINO/Ag, as demonstrated in Figure 2a. A 1.2:1 donor-to-acceptor ratio, a 0.5% (*v*/*v*) additive of 1-chloronaphthalene (1-CN), an annealing temperature of 110 °C, and a rotation speed of 2500 rpm/40 s were the ideal preparation conditions for the optimized device. The *J-V* curve, which was produced after optimizing the device settings, is shown in Figure 2b and Table 2, where the data acquired are presented in detail. The values of *V*_OC_, *J*_SC_, and FF increase from 0.71 V, 8.08 mA/cm^2^, and 42.16% to 0.80 V, 10.64 mA/cm^2^, and 49.72%, respectively, as the series progresses from **S1** to **S3**. This suggests that the performance of OSC devices can be positively impacted by reasonable molecular design, specifically the addition of an alkyl chain and the thiophene π-bridge, since the PCE of **S3** (4.25%) is nearly 1.8 times higher than that of **S1** (2.42%). It should be mentioned that **S3** has a smaller band gap (1.69 eV) and a lower HOMO energy level (−5.20 V), so the **S3**:**Y6** device has a higher *V*_OC_ (0.80 V). On the other hand, adding an alkyl chain improves the solubility of the small donor molecule material, which in turn improves the morphology of the device film and leads to better *J*_SC_ (10.64 mA/cm^2^) and FF (49.52%). Figure 2c shows that the three small donor molecules all have a strong light absorption from 480 nm to 830 nm in the 300–950 nm range of the external quantum efficiency (EQE) curve. This suggests that adding thiophene as a π-bridge and a sufficiently long alkyl chain to it can enhance the light absorption response intensity of the small donor molecule materials. Each and every integrated *J*_SC_ that was estimated by utilizing the EQE curves is in agreement with the *J*_SC_ that was measured, and this agreement is within the error range by a margin of less than five percent.

**Figure 3:**
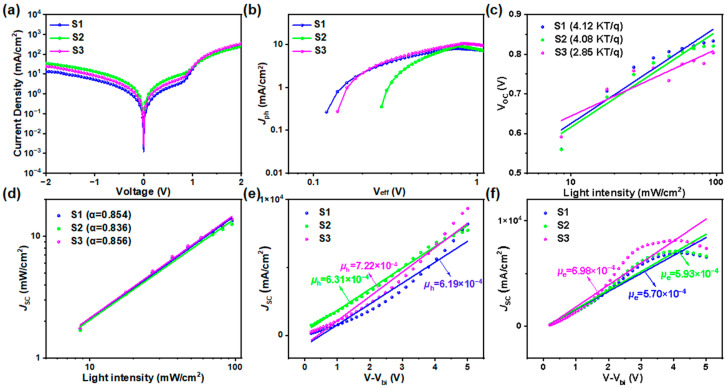
(**a**) Semilogarithmic plot of *J*-*V* characteristics of devices in the dark; (**b**) *J*_ph_-*V*_eff_ characteristics; (**c**) *V*_OC_ and (**d**) *J*_SC_ versus light intensity of the OSCs; (**e**) *J*-*V* curves of the hole-only device; (**f**) *J*-*V* curves of the electron-only device.

Using the *J-V* characteristic curve in dark conditions, the charge transfer process in OSCs devices is examined, as illustrated in Figure 3a. The device based on **S3**:**Y6** exhibits the highest dark current density in the presence of a positive bias. Conversely, when the bias is negative, the dark current density is in the middle. The device based on **S3**:**Y6** possesses a more asymmetrical *J-V* characteristic curve, which makes charge extraction and collection easier [69]. Additionally, it inhibits current leakage, resulting in a higher *J*_SC_ (10.64 mA/cm^2^). Based on the findings presented in Figure 3b, the photogenerated current density profile and the effective voltage curve (*J*_ph_ − *V*_eff_) were utilized in order to investigate the charge extraction and exciton dissociation capabilities of OSCs. Among them, *J*_ph_ = *J*_L_ − *J*_D_, where *J*_L_ and *J*_D_ are the current densities under 100 mW cm^−2^ light and darkness, and *V*_eff_ = *V*_0_ − *V*_a_, where *V*_0_ is the voltage when *J*_ph_ is zero and *V*_a_ is the applied voltage. The devices that were based on **S3**:**Y6** had a greater exciton dissociation efficiency, which resulted in increased hole and electron mobility. This was in contrast to the devices that were based on **S1**:**Y6**, **S2**:**Y6**, and **S3**:**Y6** organic semiconductor capacitors, which had dissociation efficiencies of 46.74%, 58.36%, and 62.91%, respectively.

As shown in Figure 3c,d, the charge recombination mode in OSCs was studied through the curve of *V*_OC_ and *J*_SC_ with light intensity. The curve fitting between *V*_OC_ and light intensity was *V*_OC_∝n(K_B_T/q)lnP_light_. When n = 1, it indicated that only bimolecular recombination existed in the device; when n = 2, it indicated that trap-assisted recombination was dominant in the device. The slope of the **S3**:**Y6** device is 2.85 KT/q, which is much lower than that of the **S2**:**Y6** device (4.08 KT/q), indicating that the introduction of alkyl chain can effectively inhibit the trap-assisted recombination behavior based on small donor molecule devices. Similarly, the slope of the **S2**:**Y6** device is smaller than that of the **S1**:**Y6** device (4.12 KT/q), indicating that the introduction of thiophene π-bridge plays a weak positive role in effectively inhibiting the trap assist behavior in the device. The curve fitting between *J*_SC_ and light intensity was *J*_SC_∝P_light_α. The α values of the **S1**:**Y6**, **S2**:**Y6**, and **S3**:**Y6** OSCs devices were 0.854, 0.836, and 0.856, respectively. These α values were similar, indicating that they all had similar weak bimolecular recombination behavior.

As shown in Figure 3e,f, hole mobility (*μ*_h_) and electron mobility (*μ*_e_) of the device were measured by the SCLC method to study charge transport in the device, and the detailed data are shown in Table 3. The device with pure hole (ITO/PEDOT:PSS/Donor:**Y6** (1.2:1)/MoO_3_/Ag) structure has been prepared to measure *μ*_h_ and pure electron (ITO/ZnO/Donor:**Y6** (1.2:1)/PDINO/Ag) structure of the device measurement of *μ*_e_. Based on **S1**:**Y6**, **S2**:**Y6**, and **S3**:**Y6** OSCs devices, *μ*_h_ and *μ*_e_ are 6.19 × 10^−4^ cm^2^ V^−1^ s^−1^/5.70 × 10^−4^ cm^2^ V^−1^ s^−1^ (*μ*_h_/*μ*_e_ = 1.09), 6.31 × 10^−4^ cm^2^ V^−1^ s^−1^/5.93 × 10^−4^ cm^2^ V^−1^ s^−1^ (*μ*_h_/*μ*_e_ = 1.06), and 7.22 × 10^−4^ cm^2^ V^−1^ s^−1^/6.98 × 10^−4^ cm^2^ V^−1^ s^−1^ (*μ*_h_/*μ*_e_ = 1.03), respectively. It can be seen from the data that the device based on **S3**:**Y6** has the highest and balanced *μ*_h_ and *μ*_e_, so, based on the **S3**:**Y6** device, it has a higher FF.

## 3. Materials and Methods

### 3.1. Materials

The BDT-2SnMe_3_ was acquired from SunaTech Inc (SooChow, China), while the non-fullerene acceptor **Y6** was acquired from Shuo Lun Organic Optoelectronic Technology (Jiaxing, China). The electronic transport layer was composed of PDINO, which was acquired from Shanghai Dusen Optoelectronic Materials (Shanghai, China), and the hole transport layer was composed of PEDOT:PSS (4083) from Xi’an Baolaite Optoelectronic Technology (Xi’an, China). Unless otherwise specified, all of the other drugs and reagents that were utilized in the synthesis were acquired from Shanghai Anneji Chemical (Shanghai, China). These pharmaceuticals and reagents can be utilized without the need for additional purification. Chromatoform, methylethanol, zinc acetate dihydrate, and other solvents were purchased from Sigma-Aldrich (St. Louis, MO, USA) in order to be utilized in the process of preparing the ZnO electronic transport layer solution. After treating with calcium hydride, anhydrous chloroform and toluene can be obtained through distillation.

### 3.2. Characterization

The Bruker 400 and 600 spectrometers (Bruker, Karlsruhe, Germany) were utilized in order to acquire the ^1^H NMR spectrum in CDCl_3_. The cyclic voltammetry (CV) was performed with the help of a CHI661C electrochemical workstation, and the UV-vis absorption spectra were analyzed with a Shimadzu UV-2450 spectrophotometer (UV-2600, SHIMADZU, Kyoto, Japan). The CV curve calibration was carried out using an external reference, a ferrocene/ferrocenium (Fc/Fc^+^) redox couple, and a carbon glass electrode covered with **S1**, **S2**, or **S3** films. The working electrode was placed in a 0.1 mol/L acetonitrile solution of tetrabutylammonium phosphorus hexafluoride (Bu_4_NPF_6_). The reference electrode was an Ag/Ag^+^ electrode, and the working electrode was a carbon glass electrode.

### 3.3. Synthesis of Compound ***1*** (DTP46-CHO)

A reaction tube with a capacity of 25 milliliters was initially filled with DMF (90.27 mg, 1.2351 mmol, 1.2 equiv.) and POCl_3_ (189.40 mg, 1.2351 mmol, 1.2 equiv.) in an atmosphere of argon. The reaction tube was then stirred at 0 ℃ for 4 h after dissolving DTP46 (300 mg, 1.0293 mmol) in DCE (2.64 mg, 26.6580 mmol, 25.9 equiv.) and was stirred for 10 min. Then, for the quenching step, we filled the reaction tube with 5 mL of saturated potassium acetate water, stirred it, and let it sit at room temperature for 2 h. After that, we used CH_2_Cl_2_ and saturated salt water to extract the reaction liquid. Next, we spun the organic phase that had been purified on a rotary evaporator with a 3:1 ratio of dichloromethane or petroleum ether. Thin-layer chromatography was used to purify the dark green viscous compound **1**, which had a mass of 258.5 mg and a yield of 78.6%. ^1^H NMR (400 MHz, CDCl_3_): δ 9.82 (s, 1H), 7.57 (s, 1H), 7.33 (d, *J* = 5.4 Hz, 1H), 6.96 (d, *J* = 5.4 Hz, 1H), 4.03 (dd, *J* = 7.3, 3.5 Hz, 2H), 1.30 (ddd, *J* = 18.4, 8.8, 3.8 Hz, 9H), and 0.94–0.81 (m, 6H).

### 3.4. Synthesis of Compound ***2*** (Br-DTP46-CHO)

To a 7 mL anhydrous CHCl_3_ solution, 250.00 mg of Compound **1** and 139.28 mg of NBS, each with a molecular weight of 0.7825 mmol, were added, making up 1 equivalent. Deionized water was used to quench the reaction after the reaction tube had been wrapped in tin foil and stirred at room temperature for 5.5 h while at a dark reaction. Using CH_2_Cl_2_ as an extractant, the raw material was isolated in saturated salt water. After subjecting the organic phase to steaming on a rotary evaporation apparatus, compound **2**, a solid with a dark green color and a yield of 70.5% (219.8 mg), was isolated and purified using thin-layer chromatography with an eluent ratio of 1:1 between dichloromethane and petroleum ether. ^1^H NMR (400 MHz, CDCl_3_) δ: 9.85 (s, 1H), 7.58 (s, 1H), 7.03 (s, 1H), 4.02 (dd, *J* = 7.4, 4.8 Hz, 2H), 1.37–1.21 (m, 9H), and 0.93–0.83 (m, 6H).

### 3.5. Synthesis of Compounds ***3*** and ***4***

Compound **2** (300.00 mg, 0.7530 mmol), 2-(tributyltin) thiophene (281.03 mg, 0.7530 mmol, 1 equiv.), and Pd(PPh_3_)_4_ (43.51 mg, 0.0377 mmol, 0.05 equiv.) were added to a Schlenk reaction tube that was 25 mL in volume. The reaction tube was then vacuumed alternately at a low temperature of 0 °C, and filled with argon to remove any air. Following this, 5 mL of anhydrous toluene was added to the reaction tube, and oxygen in the reaction tube was removed three times through a pumping air-filled argon cycle. Finally, the mixture was stirred at a temperature of 100 °C under an atmosphere of argon. The final step in the reaction was to extract the crude product by evaporating the solvent toluene. The third compound, a yellow-green solid, was isolated by thin-layer chromatography from 227.6 mg of a mixture of dichloromethane and petroleum ether in a ratio of 1:1. The yield was 70.5%. Chemical compound **4**, which has a yellow-green solid state and a yield of 63.0% (270.4 mg), was also produced using the same procedure.

**Compound 3:** ^1^H NMR (400 MHz, CDCl_3_) δ: 9.84 (s, 1H), 7.56 (s, 1H), 7.28 (d, *J* = 0.9 Hz, 1H), 7.25 (s, 1H), 7.06–7.01 (m, 2H), 4.05 (dd, *J* = 7.5, 4.2 Hz, 2H), 1.34–1.27 (m, 9H), and 0.93–0.87 (m, 6H).

**Compound 4:** ^1^H NMR (400 MHz, CDCl_3_) δ: 9.85 (s, 1H), 7.58 (s, 1H), 7.06 (d, *J* = 1.2 Hz, 1H), 7.01 (s, 1H), 6.84 (s, 1H), 4.07 (dd, *J* = 7.4, 4.8 Hz, 2H), 1.30 (dt, *J* = 12.3, 6.8 Hz, 28H), and 0.93–0.86 (m, 12H).

### 3.6. Synthesis of Compounds ***5*** and ***6***

During the reaction, compound **3** (200.00 mg, 0.4980 mmol) and NBS (88.63 mg, 0.4980 mmol, 1 equiv.) were added to 10 mL of anhydrous CHCl_3_ solution. The reaction tube was then wrapped in tin foil for 4.5 h at room temperature, and the reaction was finally quenched with deionized water. An extractant called CH_2_Cl_2_ was used to extract the crude product from saturated salt water. Next, the organic phase that had been purified was heated in a rotary evaporation apparatus. Then, compound **5**, a solid yellow substance with a mass of 187.8 mg and a yield of 78.5%, was isolated using thin-layer chromatography with an eluent ratio of 1:1 between dichloromethane and petroleum ether. In the same way, compound **6**, a solid yellow colorant, was produced (145.0 mg, yield 63.7%).

**Compound 5:** ^1^H NMR (400 MHz, CDCl_3_) δ: 9.83 (s, 1H), 7.55 (s, 1H), 7.06–6.90 (m, 3H), 4.11–3.96 (m, 2H), 1.30 (dd, *J* = 13.9, 6.4 Hz, 9H), and 0.88 (dt, *J* = 6.9, 4.3 Hz, 6H).

**Compound 6:** ^1^H NMR (400 MHz, CDCl_3_) δ: 9.85 (s, 1H), 7.58 (s, 1H), 7.01 (s, 1H), 6.84 (s, 1H), 4.08 (td, *J* = 7.5, 4.1 Hz, 2H), 1.35–1.23 (m, 26H), and 0.98–0.81 (m, 14H).

### 3.7. Synthesis of Compounds ***7***, ***8***, and ***9***

The following ingredients were added to a 25 mL Schlenk reaction tube: BDT-2SnMe_3_ (100.00 mg, 0.0964 mmol), compound **2** (84.47 mg, 0.2120 mmol, 2.2 equiv.), and Pd(PPh_3_)_4_ (5.57 mg, 0.0048 mmol, 0.05 equiv.). The tube was then vacuumed alternately at low temperature and filled with argon to remove air and 5 mL of anhydrous toluene was added. Oxygen was removed from the reaction tube by pumping the air-filled argon cycle three times. The mixture was then stirred at 100 °C under an argon atmosphere for 48 h. When the reaction was complete, the solvent toluene was evaporated in order to yield a crude product, which has a ratio of dichloromethane to petroleum ether that is equal to two to one. Thin-layer chromatography was utilized in order to purify the red solid chemical **7** (79.9 mg, yield 61.7%). The identical procedure was used to produce red solid compounds **8** and **9**, with respective yields of 52.4% and 48.8%. The dosages of these compounds were 76.2 mg and 86.8 mg.

**Compound 7:** ^1^H NMR (400 MHz, CDCl_3_) δ: 9.69 (s, 2H), 7.45 (s, 2H), 7.34 (d, *J* = 14.9 Hz, 4H), 6.96 (s, 2H), 4.04 (dd, *J* = 16.4, 5.4 Hz, 4H), 3.04 (d, *J* = 6.1 Hz, 4H), 1.55 (dt, *J* = 19.8, 6.4 Hz, 10H), 1.32 (ddd, *J* = 22.9, 11.9, 7.9 Hz, 30H), and 1.00–0.90 (m, 20H).

**Compound 8:** ^1^H NMR (400 MHz, CDCl_3_) δ: 9.65 (s, 2H), 7.38–7.27 (m, 6H), 6.98 (t, *J* = 15.6 Hz, 4H), 6.78 (d, *J* = 25.2 Hz, 2H), 3.96 (s, 4H), 3.06 (dd, *J* = 15.6, 5.8 Hz, 4H), 1.62–1.51 (m, 10H), 1.34–1.24 (m, 21H), and 0.92 (ddd, *J* = 15.7, 10.0, 5.6 Hz, 20H).

**Compound 9:** ^1^H NMR (400 MHz, CDCl_3_) δ: 9.65 (s, 2H), 7.34 (s, 4H), 7.00 (d, *J* = 7.9 Hz, 4H), 6.81 (s, 2H), 3.96 (s, 4H), 3.04 (d, *J* = 6.1 Hz, 4H), 1.38–1.25 (m, 34H), and 1.00–0.89 (m, 26H).

### 3.8. Synthesis of ***S1***, ***S2***, and ***S3***

In a round-bottom flask, compound **7** (50.00 mg, 0.0372 mmol), RDN-34 (42.25 mg, 0.2232 mmol, 6 equiv.), 2.5 mL of anhydrous toluene, 50 μL of acetic anhydride, and 50 μL of BF_3_-OEt_2_ were added. To determine whether or not the reaction of the raw materials is adequate, a capillary tube is used to perform the sampling point plate of the reaction liquid at any point in time throughout the reaction process. Following the evaporation of the solvent toluene at the conclusion of the reaction, which results in the formation of a crude product, dichloromethane/petroleum ether = 2:1 is used as the eluent, and the blue-violet solid substance **S1** (40.1 mg, yield 63.8%) is purified using thin-layer chromatography. The same procedure was used to manufacture the blue-violet solid substance **S2** (34.8 mg, yield 56.6%) and the blue-violet solid compound **S3** (29.3 mg, yield 63.8%) with the same results.

**S1:** ^1^H NMR (400 MHz, CDCl_3_) δ: 7.44 (s, 2H), 7.29 (s, 2H), 7.10 (s, 2H), 6.77 (d, *J* = 39.1 Hz, 4H), 3.93 (s, 8H), 3.09 (d, *J* = 6.0 Hz, 4H), 1.65–1.51 (m, 14H), 1.47–1.25 (m, 32H), and 0.97 (tdd, *J* = 22.3, 14.8, 7.4 Hz, 32H).

**S2:** ^1^H NMR (400 MHz, CDCl_3_) δ: 7.78–7.28 (m, 4H), 7.14–6.44 (m, 10H), 3.80 (d, *J* = 35.4 Hz, 8H), and 1.66–0.87 (m, 78H).

**S3:** ^1^H NMR (400 MHz, CDCl_3_) δ: 7.84 (s, 2H), 7.55 (s, 2H), 7.35 (s, 2H), 7.16 (s, 2H), 7.00 (d, *J* = 21.2 Hz, 4H), 4.14–4.00 (m, 8H), 3.01 (d, *J* = 6.1 Hz, 4H), 1.57–1.49 (m, 8H), 1.32–1.22 (m, 40H), and 0.87 (ddd, *J* = 20.1, 10.0, 4.5 Hz, 30H).

### 3.9. Device Fabrication

The indium tin oxide (ITO)-coated glass was pre-cleaned in an ultrasonic bath with deionized water, acetone, and ethanol multiple times after the BHJ OPV devices based on the ITO/PEDOT:PSS/active layer/PDINO/Ag structure were prepared. The next step was a five-minute plasma treatment of the glass. On the ITO substrates, the PEDOT:PSS layer was spin-coated at a speed of 2500 revolutions per minute for forty seconds. After that, the layer was annealed at a temperature of 110 degrees Celsius for ten minutes. By dissolving Donor:**Y6** (1.2:1, weight%) in CF at a concentration of 17.6 mg/mL, as well as adding 0.5% 1-CN and agitating the mixture for more than 30 min, an active layer solution was created. After the spin-coated active layer underwent thermal annealing in a nitrogen glove box, a 0.043 cm^2^ area was vacuum-deposited with 10 nm PDINO and 100 nm Ag. Under a monochromatic-lit 150 W xenon lamp with a Cornerstone 74004 lens, we measured the external quantum efficiency (EQE), and we analyzed the J-V curves in a glovebox with an AM 1.5 G solar spectrum (100 mW/cm^2^).

## 4. Conclusions

In this study, three different small donor molecule materials were synthesized using the Vilsmeier–Haack reaction, classical Stille coupling, and Knoevenagel condensation. These approaches were utilized to construct the final target small donor molecule materials (**S1**, **S2**, and **S3**), which were then analyzed using UV-*vis*, CV, *J-V*, and SCLC. The incorporation of a thiophene π-bridge into **S2** from **S1** enhances the blue shift of UV absorption, narrows the band gap, and lowers the HOMO energy level. As a consequence, the *V*_OC_ of **S2**:**Y6** devices increases to 0.75 V, and a 3% PCE is achieved. **S3**, which was created by adding an alkyl chain to a thiophene π-bridge, outperforms **S2** in terms of solubility and π-π stacking, leading to enhanced exciton dissociation and charge collection capabilities following device preparation. Additionally, after blending with **Y6**, the highest *J*_SC_ (10.59 mA/cm^2^), FF (49.72%), and PCE (4.25%) were achieved. According to recent studies, when designing molecules for molecular simulation, it is important to first check if the length of the alkyl chain can guarantee enough solubility. If it cannot, then the molecular accumulation during device preparation will be too strong, which will impact charge transport and make it impossible to harvest more effective current and voltage, ultimately hindering the device’s efficiency. The incorporation of an alkyl chain into the thiophene π-bridge and a thiophene π-bridge can enhance the device’s morphology and charge transfer behavior. This could be a promising strategy for the future design and synthesis of materials including tiny donor molecules.

## Figures and Tables

**Table 1:** Optical and electrochemical data of **S1**, **S2**, and **S3**.

	λ_max_ *^s^* (nm) *^a^*	λ_max_ *^f^* (nm) *^b^*	λ_onset_ (nm) *^b^*	E_g_ (eV) *^c^*	E_HOMO_ (eV) *^d^*	E_LUMO_ (eV) *^d^*
**S1**	591	580	708	1.75	−5.14	−3.61
**S2**	580	585	724	1.71	−5.18	−3.68
**S3**	595	586	732	1.69	−5.20	−3.72

*^a^* Measured in chloroform solutions. *^b^* Cast from chloroform solutions. *^c^* Optical band gaps estimated from the onset wavelength (λ_onset_): E_g_ = 1240/λ_edge_. *^d^* Measured by CV. *^s^* In CHCl_3_ solution. *^f^* In thin films.

**Table 2:** Photovoltaic performance of the device by ITO/PEDOT:PSS/Donor:**Y6**/PDINO/Ag.

	*V*_OC_ (V)	*J*_SC_ (mA/cm^2^)	*J*_SC_ *^a^* (mA/cm^2^)	FF (%)	PCE *^b^* (%)
**S1:Y6**	0.71 (0.70 ± 0.005)	8.08 (7.46 ± 0.48)	7.93	42.16 (41.25 ± 0.89)	2.42 (2.27 ± 0.14)
**S2:Y6**	0.75 (0.75 ± 0.004)	9.23 (8.91 ± 0.33)	9.20	42.93 (42.59 ± 0.46)	3.00 (2.85 ± 0.11)
**S3:Y6**	0.80 (0.79 ± 0.002)	10.64 (10.17 ± 0.51)	10.59	49.72 (49.62 ± 0.39)	4.25 (4.21 ± 0.07)

The values in brackets are the average values and standard deviations. *^a^* Integral *J*_SC_ from EQE curves. *^b^* Average PCEs based on 20 independent devices.

**Table 3:** Based on **S1**:**Y6**, **S2**:**Y6**, and **S3**:**Y6** OSCs devices for electron mobility and hole migration.

	*μ*_h_ (cm^2^ V^−1^ s^−1^)	*μ*_e_ (cm^2^ V^−1^ s^−1^)	*μ*_h_/*μ*_e_
**S1:Y6**	6.19 × 10^−4^	5.70 × 10^−4^	1.09
**S2:Y6**	6.31 × 10^−4^	5.93 × 10^−4^	1.06
**S3:Y6**	7.22 × 10^−4^	6.98 × 10^−4^	1.03

## Data Availability

The original contributions presented in the study are included in the article/Supplementary Material, further inquiries can be directed to the corresponding authors.

## Supplementary Materials

The following supporting information can be downloaded at: https://www.mdpi.com/article/10.3390/molecules29174231/s1. Figures S1–S12: ^1^H NMR spectra of all compounds.
