# Supplementary material for: Impact of Different π-Bridges on the Photovoltaic Performance of A-D-D′-D-A Small Molecule-Based Donors
Source: Molecules. 2024 Sep 6;29(17):4231. doi: 10.3390/molecules29174231 (PMC11396980; doi:10.3390/molecules29174231)
Supplement: Supplementary file 1 [file molecules-29-04231-s001.zip › molecules-3182333-supplementary.pdf]

Supporting Information

Impact of Different π-Bridges on the Photovoltaic Performance of A-D-D′-D-A Small Molecule-Based Donors

Lingjun Yang [1,†], Yu Wu [1,2,†], Pachaiyappan Murugan [1,†], Peng Liu [1], Yulong Peng [1], Zhiyong Qiu [1], Zaifang Li [2,*], Changlin Yu [3] and Shiyong Liu [1,*]

[1] Jiangxi Provincial Key Laboratory of Functional Molecular Materials Chemistry, Department of Chemistry and Chemical Engineering, Jiangxi University of Science and Technology, Ganzhou 341000, China; 17779114525@163.com (L.Y.); 18011263004@163.com (Y.W.); p.kumu29@gmail.com (P.M.); 6720221036@mail.jxust.edu.cn (P.L.); 6720230419@mail.jxust.edu.cn (Y.P.); 6720230416@mail.jxust.edu.cn (Z.Q.)

[2] China-Australia Institute for Advanced Materials and Manufacturing (IAMM), Jiaxing University, Jiaxing 314001, China

[3] Guangdong Provincial Key Laboratory of Advanced Green Lubricating Materials, Maoming 525000, China; yuchanglinjx@163.com

* Correspondence: zaifang.li@zjxu.edu.cn (Z.L.); chelsy@jxust.edu.cn or chelsy@zju.edu.cn (S.L.)

† These authors contributed equally to this work.

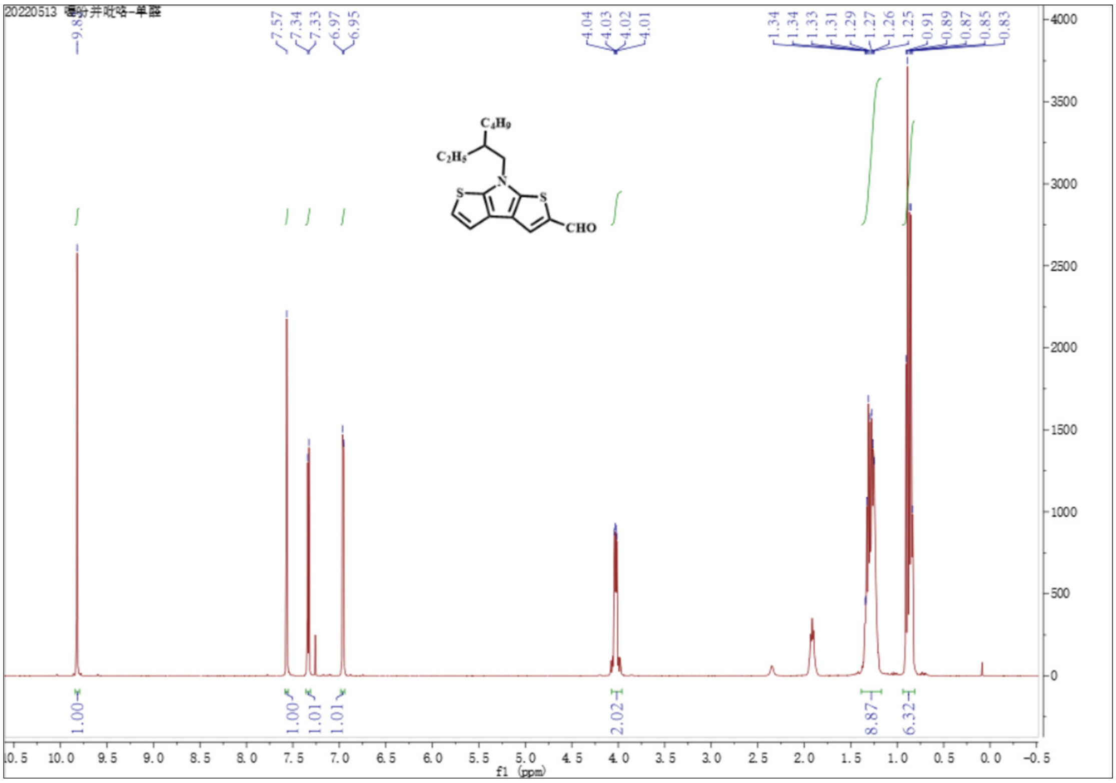

Figure S1: ^{1}H NMR spectra of compound 1 (DTP46-CHO) in $CDCl_3$.

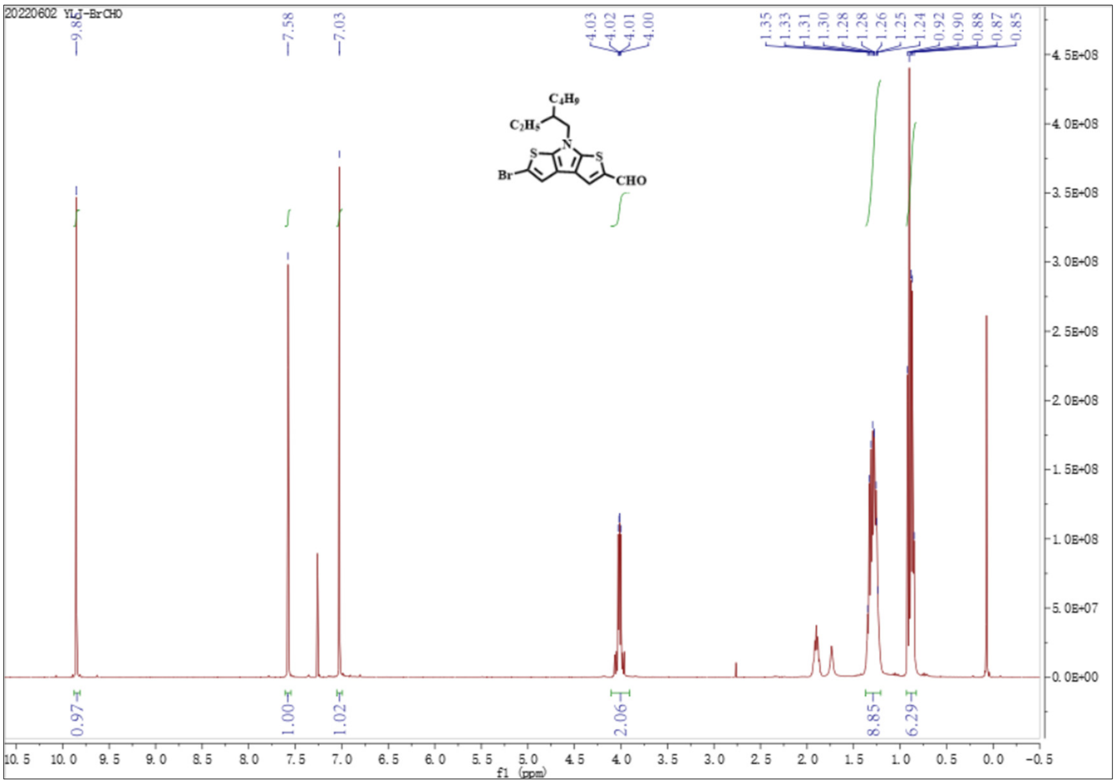

Figure S2: ^{1}H NMR spectra of compound 2 (Br-DTP46-CHO) in $CDCl_3$.

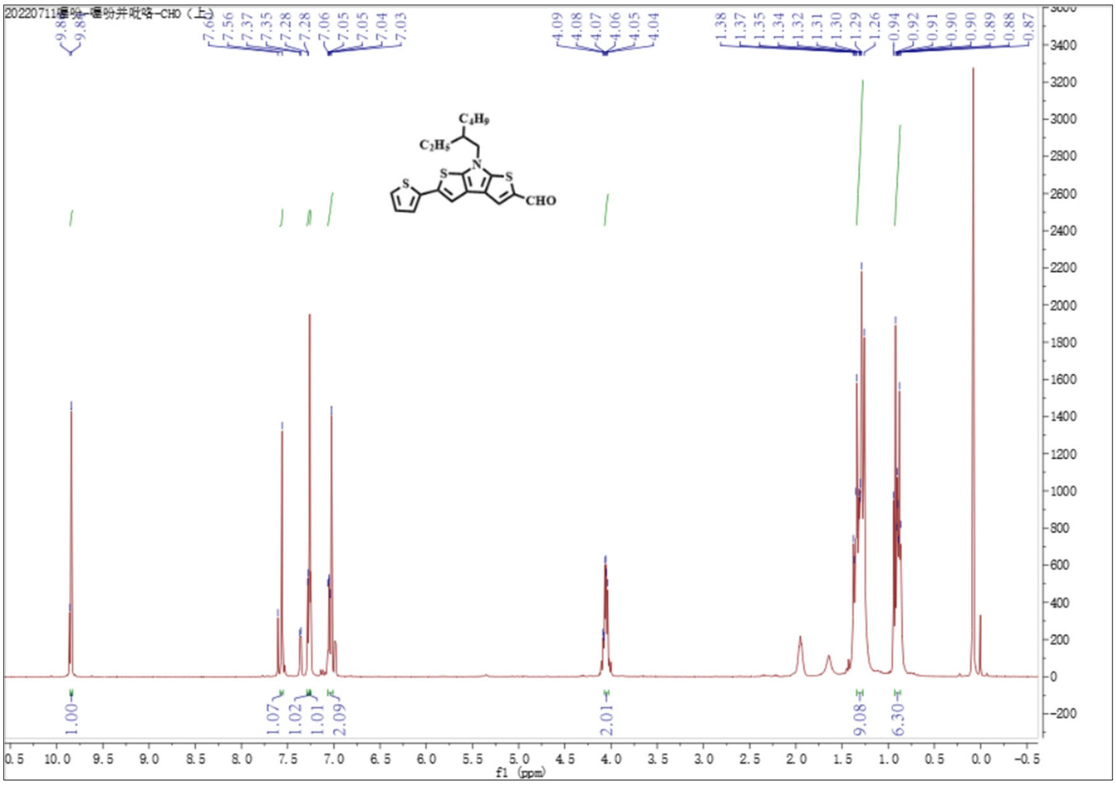

Figure S3: ^{1}H NMR spectra of compound 3 in $CDCl_3$.

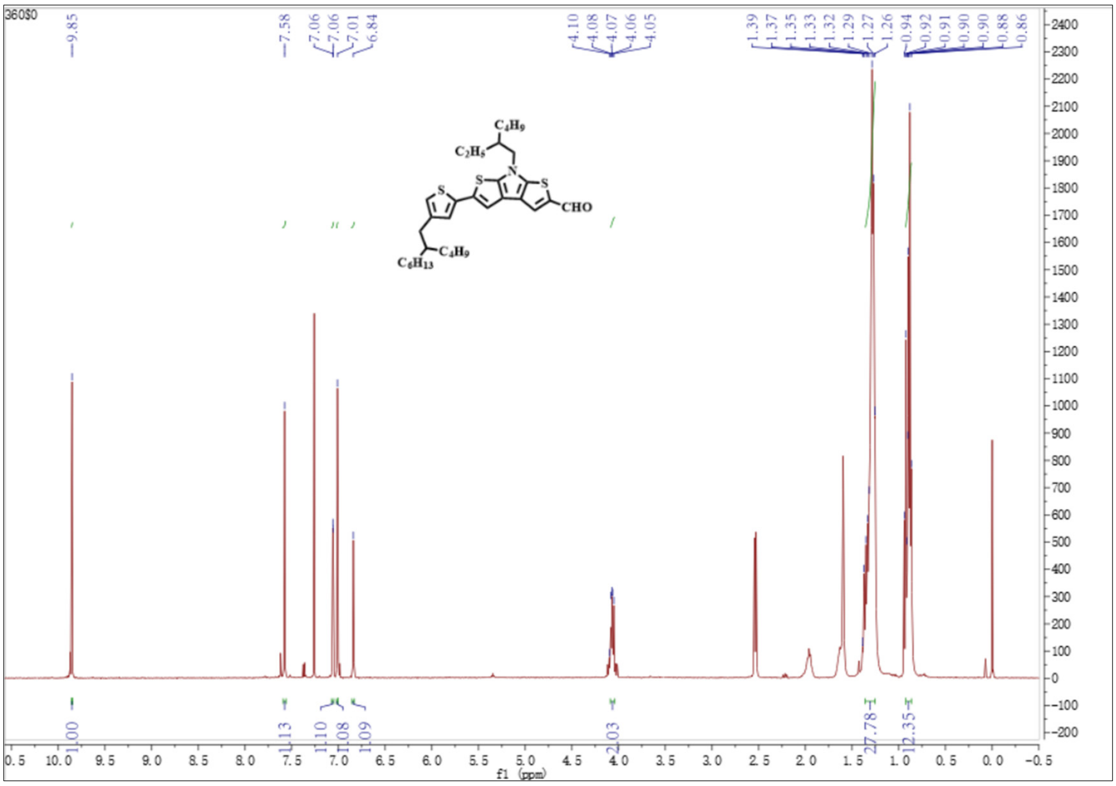

Figure S4: ^{1}H NMR spectra of compound 4 in $CDCl_3$.

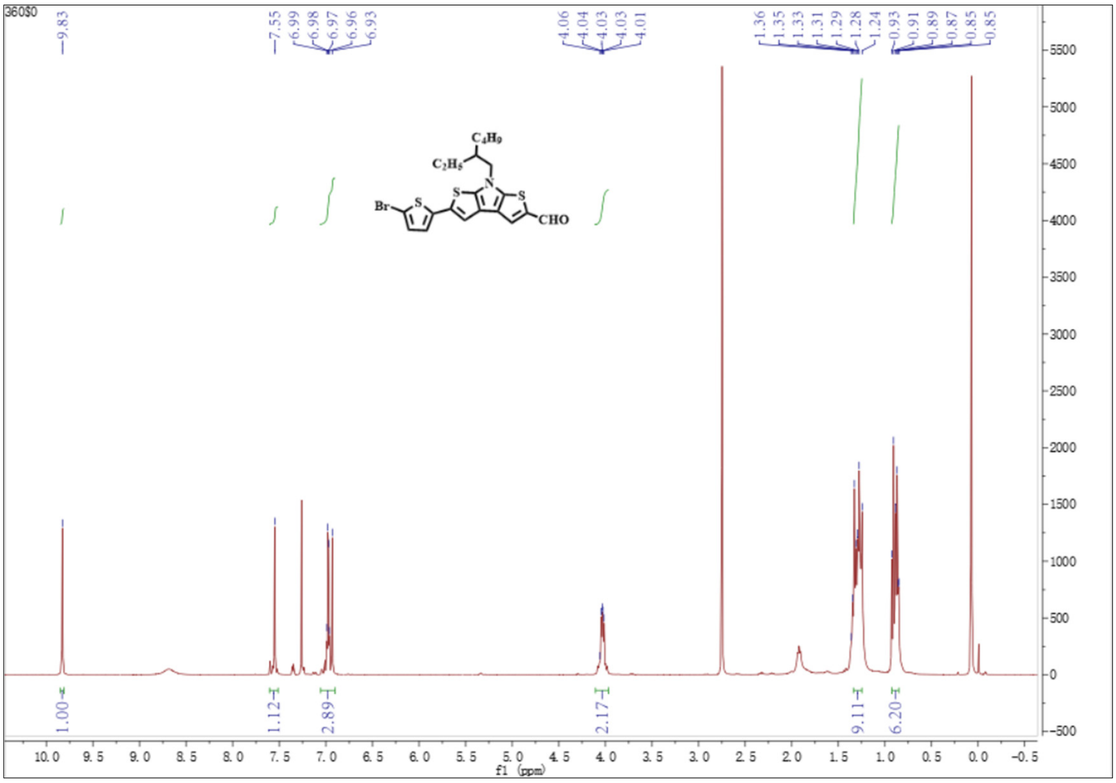

Figure S5: ^{1}H NMR spectra of compound 5 in $CDCl_3$.

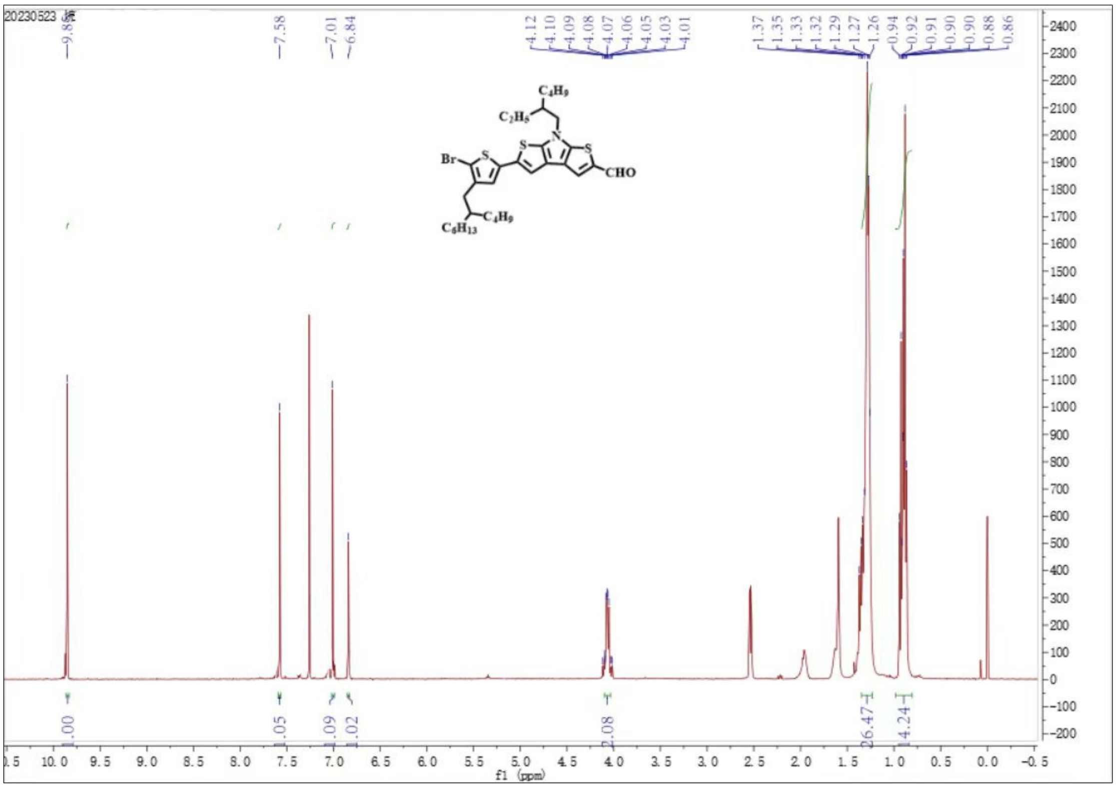

Figure S6: ^{1}H NMR spectra of compound 6 in $CDCl_3$.

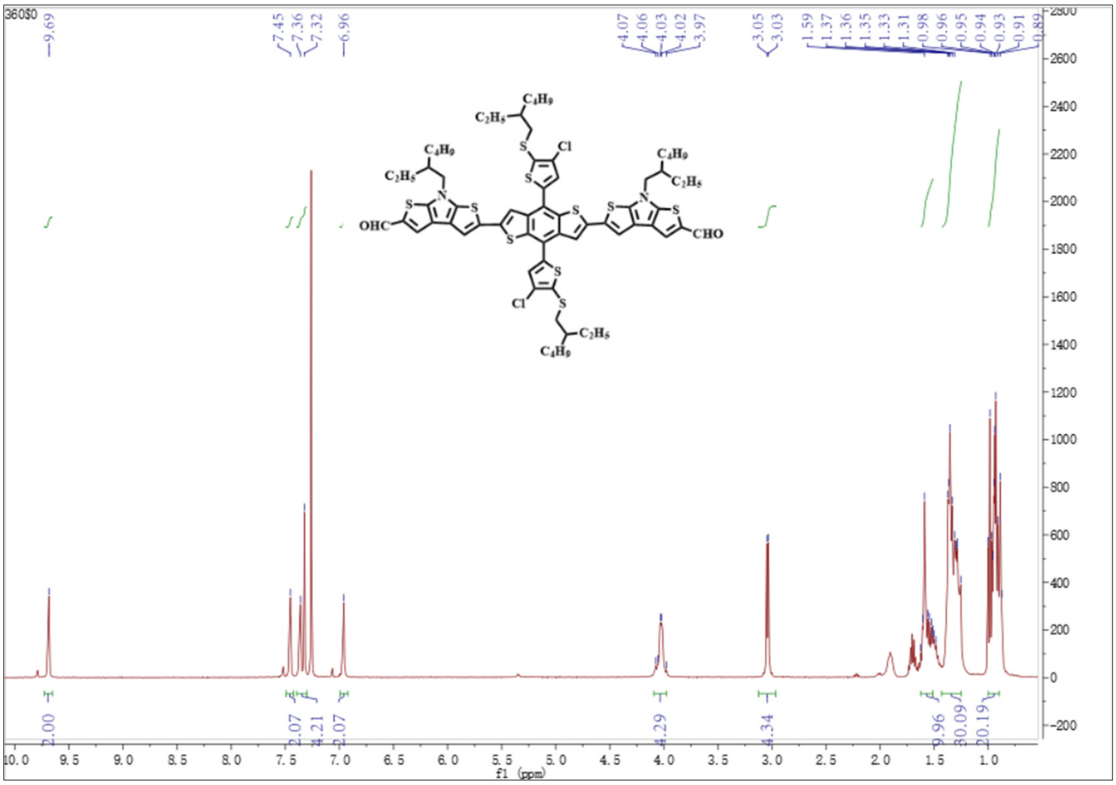

Figure S7: ^{1}H NMR spectra of compound 7 in CDCl3.

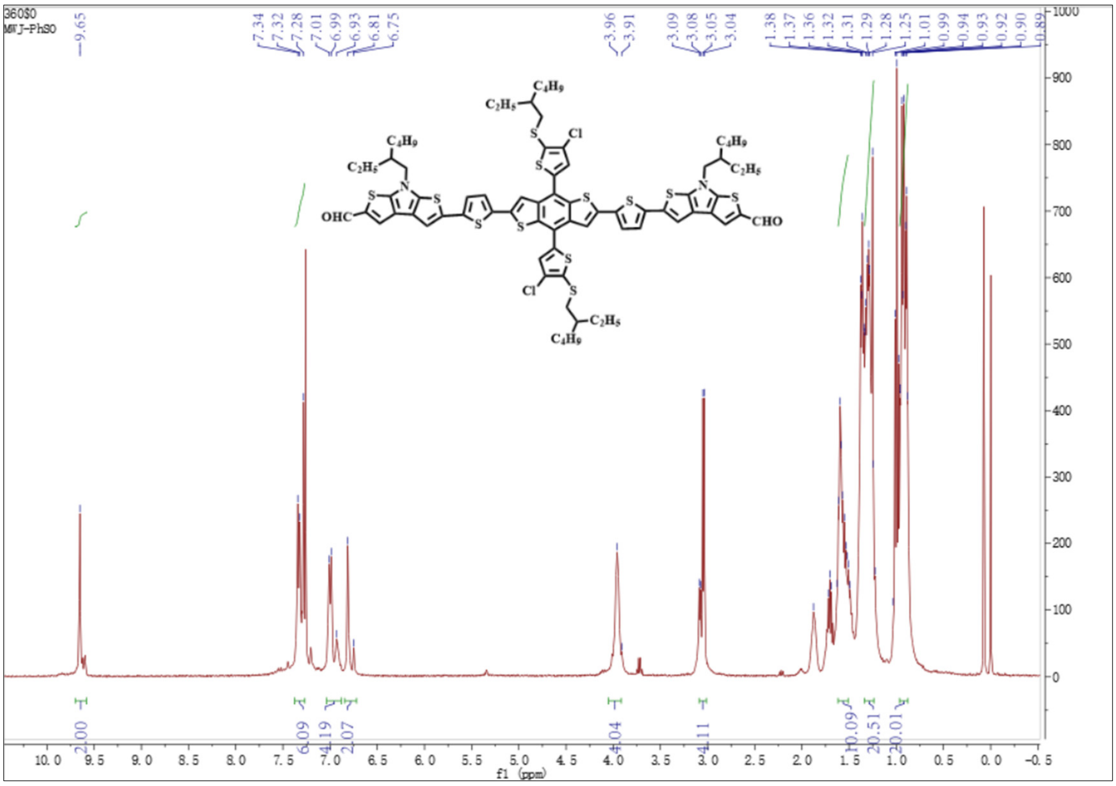

Figure S8: ^{1}H NMR spectra of compound 8 in CDCl3.

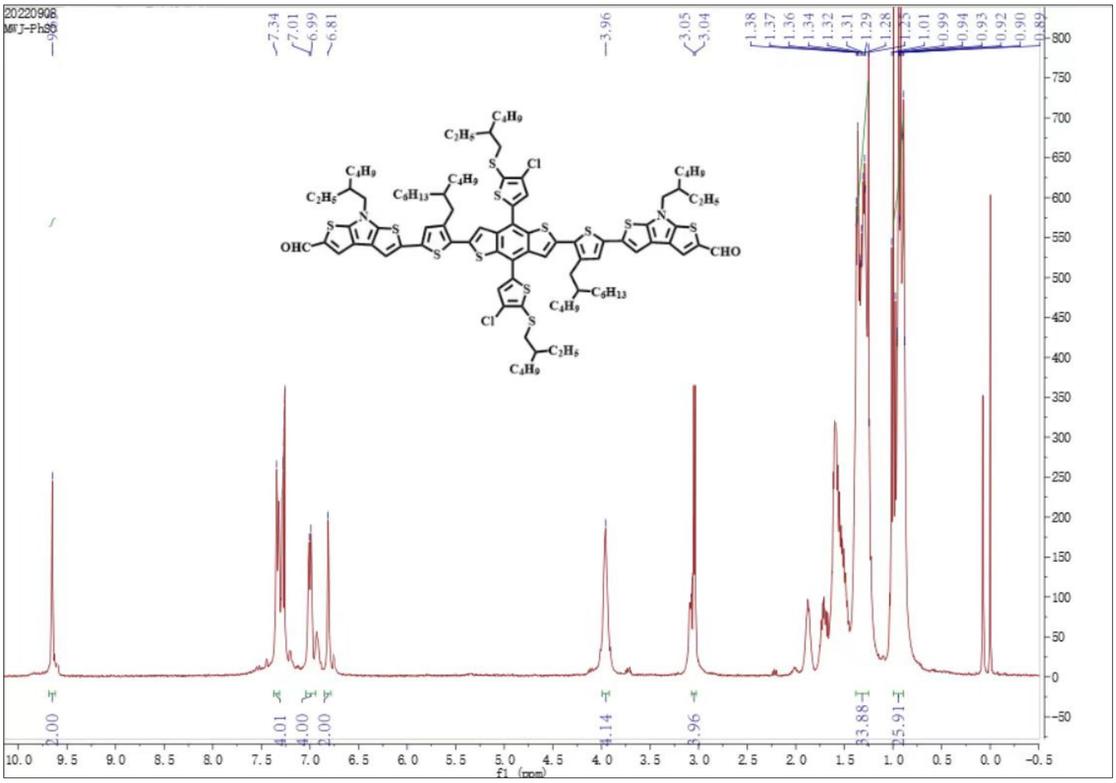

Figure S9: ^{1}H NMR spectra of compound 9 in $CDCl_3$.

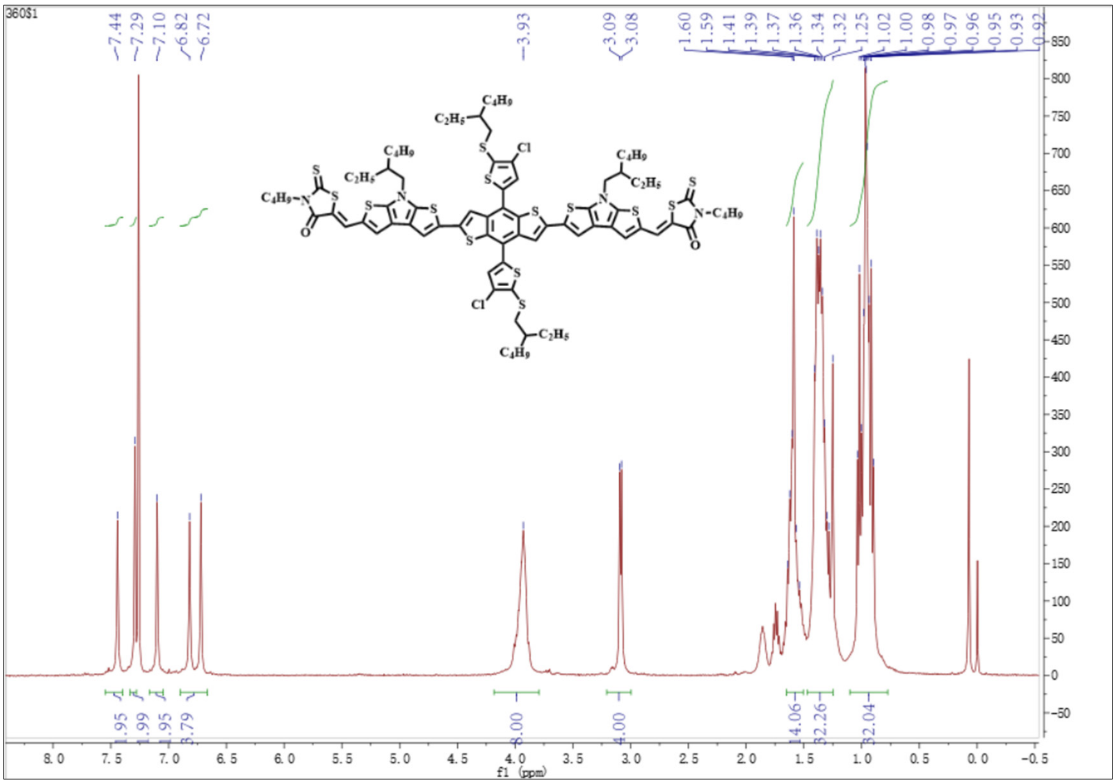

Figure S10: ^{1}H NMR spectra of S1 in $CDCl_3$.

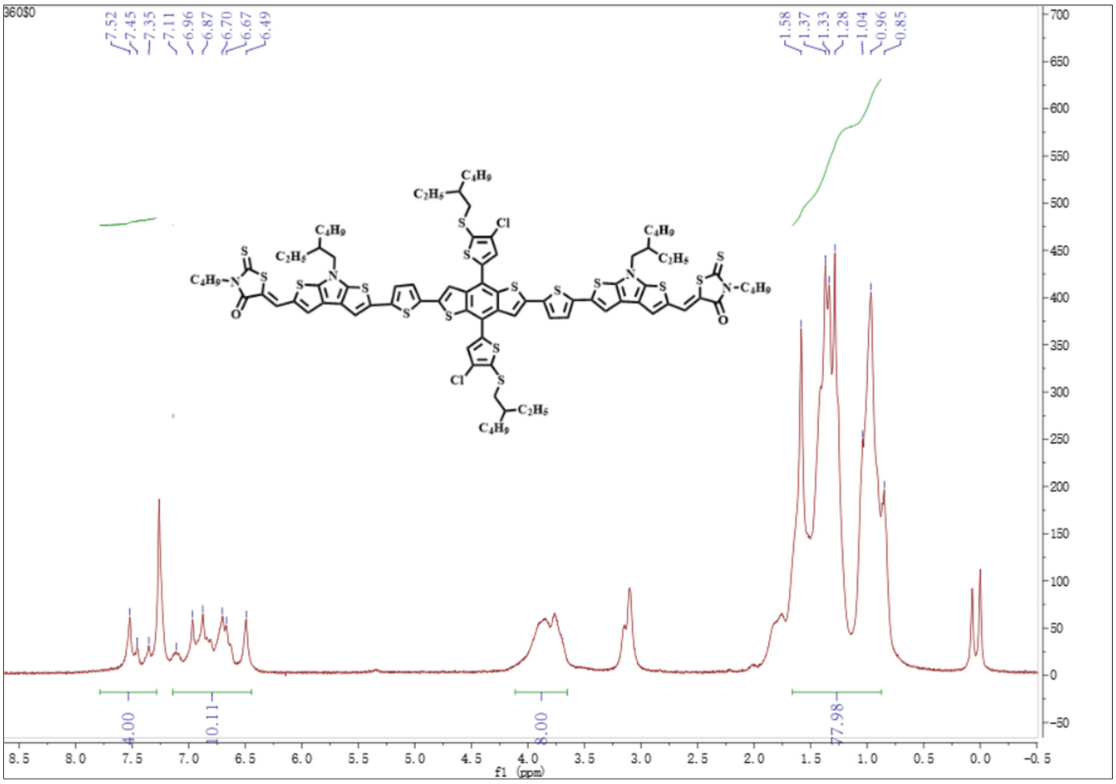

Figure S11: ^{1}H NMR spectra of S2 in $CDCl_3$.

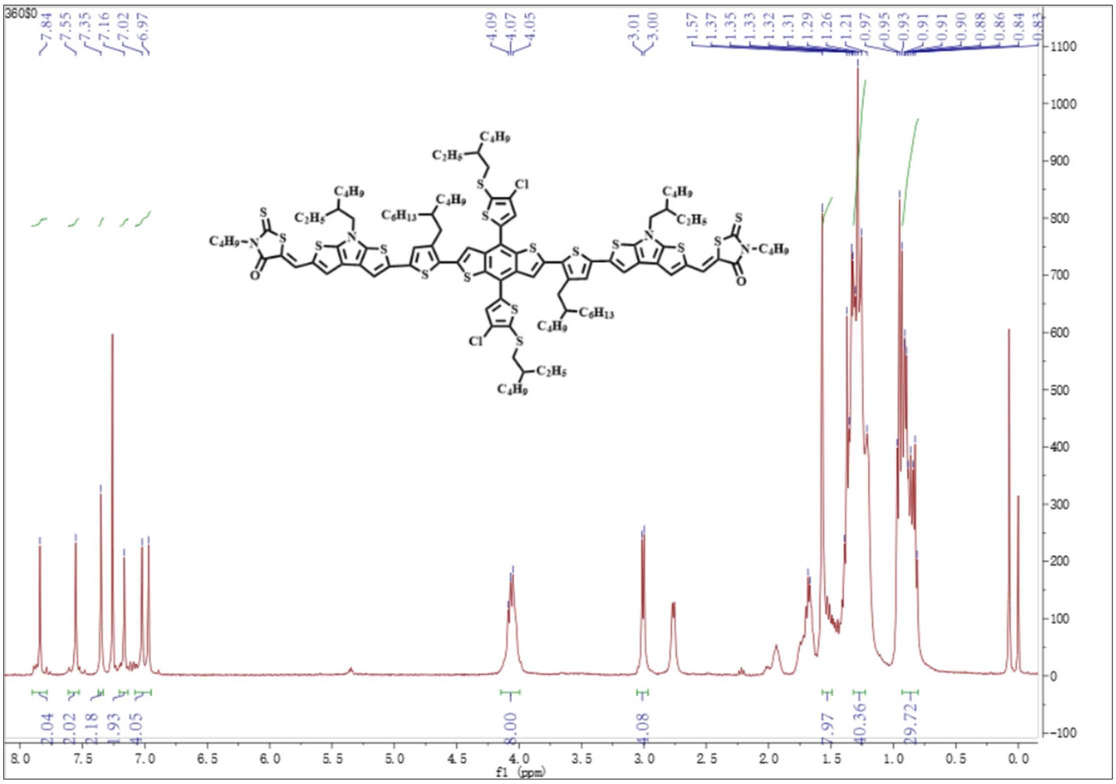

Figure S12: ^{1}H NMR spectra of S3 in $CDCl_3$.
